# Selenium Intake and its Interaction with Iron Intake Are Associated with Cognitive Functions in Chinese Adults: A Longitudinal Study

**DOI:** 10.3390/nu14153005

**Published:** 2022-07-22

**Authors:** Ke Jiang, Changxiao Xie, Zhourong Li, Huan Zeng, Yong Zhao, Zumin Shi

**Affiliations:** 1 School of Public Health, Chongqing Medical University, Chongqing 400016, China; 2021110631@stu.cqmu.edu.cn (K.J.); 2019110926@stu.cqmu.edu.cn (C.X.); 2021120781@stu.cqmu.edu.cn (Z.L.); huanzeng@cqmu.edu.cn (H.Z.); 2 Research Center for Medicine and Social Development, Chongqing Medical University, Chongqing 400016, China; 3 Research Center for Public Health Security, Chongqing Medical University, Chongqing 400016, China; 4 Chongqing Key Laboratory of Child Nutrition and Health, Children’s Hospital of Chongqing Medical University, Chongqing 400014, China; 5 Human Nutrition Department, College of Health Sciences, QU Health, Qatar University, Doha 2713, Qatar; zumin@qu.edu.qa

**Keywords:** selenium intake, cognitive function, iron intake, Chinese, adults, nutrition survey

## Abstract

Studies on the relation between selenium intake and cognitive function are inconclusive. This study aimed to examine the associations between dietary selenium intake and cognitive function among Chinese adults and tested the interaction effect of selenium intake and iron intake on cognition. Data from 4852 adults aged 55 years and above who attended the 1991–2006 China Health and Nutrition Survey (CHNS) were used. Cognitive function was assessed through face-to-face interviews in 1997, 2000, 2004, and 2006. A 3-day, 24-hour recall was used to collect dietary selenium intake. Multivariable mixed linear regression and logistic regression were used in the analyses. In fully adjusted regression models, the regression coefficients (95% confidence interval) were 0.00, 0.29 (−0.12–0.70), 0.26 (−0.18–0.70), and 0.50 (0.02–0.97) across the quartiles of selenium intake. In the subgroup analysis, the positive association between selenium intake and cognitive function was only observed in the participants who live in the southern region but not those in the northern region. The selenium-intake-to-iron-intake ratio was inversely associated with low global cognition scores. Furthermore, only those with a normal BMI had a positive association between selenium and cognition. In conclusion, high selenium intake was linked to better cognitive function and a lower risk of cognition decline in Chinese adults among those with low iron intake. A substantial interaction was found between selenium intake and BMI or region.

## 1. Introduction

Dementia imposes a considerable burden on human health. This disease was reported in 2020 to occur in over 55 million people worldwide and the occurrence was reported to likely double every 20 years [1,2]. The total estimated worldwide cost of dementia was USD 1.3 trillion in 2019, which represented 1.4% of the global gross domestic product (GDP). If global dementia care was a country, it would have the 15th largest economy in the world [1,2].

Selenium is an essential trace element that protects tissues from oxidative damage [3]. It also plays a critical role in anti-inflammatory effects, thyroid hormone metabolism, DNA synthesis, antiapoptotic function, and reproduction [4]. Furthermore, selenium is vital for the central nervous system and participates in the pathology of disorders, such as Alzheimer’s disease (AD) and Parkinson’s disease (PD) [5]. The first direct evidence that selenium has a particular role in the central nervous system came from a study of children with intractable seizures who were found to have reduced glutathione peroxidase activity and showed clinical improvement after taking selenium supplements. [6]. Furthermore, selenium levels in the body decrease progressively with age and have a positive correlation with cognitive performance in older adults [7,8]. These pieces of evidence suggest that maintaining normal selenium levels in humans may reduce the risk of cognitive disorders.

However, some studies suggest that environmental exposure to excess selenium levels may cause the onset and/or development of neurobehavioral abnormalities in humans and animals [9,10]. In addition, excessive selenium exposure may impair the functions of several important proteins, neurotransmitter systems (including the dopaminergic, glutamatergic, cholinergic, and serotonergic systems), and signaling molecules involved in the management and regulation of cognitive function [9,11,12]. Population studies found that high selenium exposure is associated with the development of several neurodegenerative and neuropsychiatric diseases [13,14,15,16].

In fact, selenium deficiency and selenium overload are common. In China, 39–61% of the population have a selenium intake level below the WHO/FAO (2004) recommendation (<55 μg/day), and 10% of the population have a selenium intake level above the recommendation (>400 μg/day) [17]. Some populations may tolerate low or high selenium intake better than others (such as the Inuit in Greenland and some populations in China) [18]. In addition, previous studies focused more on Keshan disease, which is endemic to soil and grain selenium deficient areas in China [19,20]. Therefore, the relationship between selenium and cognition needs to be further explored in different populations.

Overall, studies on the association between cognitive function and selenium intake are limited. No study has examined the association between selenium intake status and cognitive function in China. This study analyzed the data from the China Health and Nutrition Survey (CHNS) to examine the associations between dietary selenium intake and cognitive function among Chinese adults aged 55 years and above to close this research gap.

## 2. Methods

### 2.1. Study Design and Study Sample

**Figure 1:**
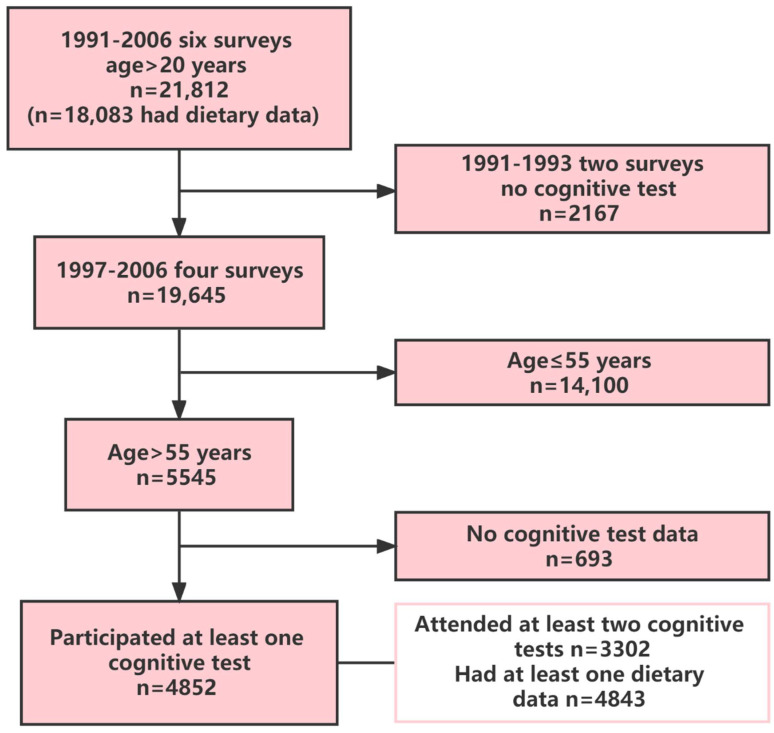
Sample flowchart of participants attending the China Health and Nutrition Survey.

The CHNS, which is an ongoing cohort survey, is a joint project between the National Institute for Nutrition and Health at the Chinese Center for Disease Control and Prevention and the Carolina Population Center at the University of North Carolina at Chapel Hill. This nationwide survey uses a multistage cluster sampling technique to select participants in urban and rural areas. A variety of methods were used for data collection, including interviews, questionnaires, physical examinations, and anthropometric measurements. Eleven waves of surveys were conducted (1989, 1991, 1993, 1997, 2000, 2004, 2006, 2009, 2011, 2015, and 2018). Cognitive function screening tests were administered to participants over the age of 55 in the surveys conducted in 1997, 2000, 2004, and 2006. A total of 4852 participants who took the cognitive screening test between 1997 and 2006 had dietary intake data (Figure 1). Among these participants, 3302 participated in ≥2 survey screening tests. Participants who completed at least one cognitive screening test were included in the analysis. The Chinese National Institute of Nutrition and Food Safety and the University of North Carolina’s Institutional Review Board both gave their approval for this study. All participants gave informed consent. The response rate of those who participated in the survey in 1989 and 2006 was more than 60%. The data used in the present study were downloaded from the CHNS website (https://www.cpc.unc.edu/projects/china. Accessed on 21 January 2022).

### 2.2. Outcome Variable: Cognitive Function

*Global cognitive function*: The cognitive function test items used in the CHNS included a subset of items from the widely used Telephone Interview for Cognitive Status-Modified (TICS-M) [21,22,23]. The test was administered through a face-to-face cognitive function screening test. The cognitive screening consisted of three tasks: (1) immediate and delayed recall of a list of 10 words (10 points each), (2) counting down from 20 to 1 (2 points), and (3) serial 7 subtraction (5 points). In the current study, the first quartile of the cognitive function test score, which is equivalent to the cutoff value < 7 for the global cognitive function score, was chosen to represent poor cognitive function. The cutoff was chosen in light of a study in Shanghai, which revealed a 20% prevalence of mild cognitive impairment in persons 60 years and older. [24].

*Self-reported memory*: Self-reported memory was assessed by the question “How is your memory? (1) Very good, (2) good, (3) OK, (4) bad, (5) very bad, (9) unknown.” Participants were defined as having a poor memory if they answered “bad” or “‘very bad”. Participants were asked about their change in memory by the question “In the past 12 months, how has your memory changed? (1) Improved, (2) stayed the same, (3) declined, (9) unknown.” Participants were defined as having self-reported memory decline if they reported “declined”.

### 2.3. Exposure Variable: Dietary Intake of Selenium

A 3-day, 24-hour recall was used to collect dietary intake data in each survey wave. During the 3-day diet survey, food and condiments from household stock, market, garden, and leftover food scraps were weighed and recorded by well-trained investigators. The intake of nutrients, including selenium, was calculated using the average of 3 days’ food consumption data in combination with the *Chinese Food Composition Table* [25]. Regional differences in the nutrient composition of foods (such as rice) grown in different regions were reflected in the *China Food Composition Table* by specific food codes. The cumulative selenium consumption was calculated and used as an exposure variable. This approach was used to reduce individual variation and reflect long-term eating patterns [26]. For instance, if a person consumed *x* in 2000, *y* in 2004, and *z* in 2006, the cumulative mean in 2004 was (*x* + *y*)/2, whereas that in 2006 was (*x* + *y* + *z*)/3. 

### 2.4. Covariates

In each survey, sociodemographic and lifestyle factors were collected using a structured questionnaire. The following constructed variables were considered covariates: age, sex, per capita annual family income (recoded as low, medium, and high), education (low, illiterate/primary school; medium, middle school; and high, senior high school and higher), urbanization levels (low, medium, and high), smoking status (non-smoker, ex-smoker, and current smoker), and leisure time physical activity (metabolic equivalent of task (MET), min/week). Hypertension was defined as systolic blood pressure over 140 mmHg, diastolic blood pressure over 90 mmHg, or known hypertension. Physical activity level (MET) was computed based on self-reported activities (including occupational, domestic, transportation, and leisure time physical activity) and duration was calculated using the Adult Compendium of Physical Activities [27]. Body mass index (BMI) was used to assess adiposity levels, and overweight was defined as BMI ≥ 24 kg/m^2^ [28]. Diabetes and stroke were self-reported and recorded as “yes” or “no”.

### 2.5. Statistical Analyses

Intake of selenium was categorized into quartiles. Frequency and proportion (%) were used to describe categorical variables, and mean ± standard deviation (SD) was used to describe continuous variables. Baseline sample characteristics were presented according to the quartiles of selenium intake and compared using a chi-square test for categorical variables or ANOVA for continuous variables. The association between cumulative mean dietary selenium intake and cognitive function was investigated using mixed-effect logistic regression analysis in Stata with the mixed command. A negative regression coefficient indicates a decline in cognitive function. To examine the association, six multivariable models were used. Model 1 was adjusted for age, gender, and energy intake. Model 2 was further adjusted for fat intake, smoking, alcohol drinking, income, urbanicity, education, and physical activity. Model 3 was further adjusted for fruit and vegetable intake. Model 4 was further adjusted for BMI and hypertension. Model 5 excluded those who only participated in one wave of the cognitive function tests. Model 6 was further adjusted for self-reported diabetes and stroke. In the subgroup analyses, the multiplicative interaction between selenium intake and covariates (income, sex, urbanization, region, overweight, and hypertension) was added by adding a product term in the regression model. The marginsplot function in Stata was utilized to visualize the interaction. Stata 17.1 (Stata Corporation, College Station, TX, USA) was used in all analyses. There was consideration of statistical significance when *p* < 0.05 (two-sided).

## 3. Results

Table 1 shows the sample characteristics of the participants who attended the first cognitive function test based on the quartiles of selenium intake. Across the quartiles of selenium intake, the mean (±SD) cumulative selenium intake was 22.3 ± 4.9, 32.5 ± 2.2, 40.3 ± 2.6, and 60.7 ± 22.9. Energy, fat, protein, carbohydrate, iron, meat, fresh vegetable, and fruit intake increased across the quartiles of selenium intake. Participants with high selenium intake had a higher BMI and were more likely to drink alcohol. Urbanization, education, and family income were positively associated with selenium intake.

### 3.1. Association between Selenium Intake and Global Cognitive Function

Selenium intake was positively associated with cognitive function (Table 2). In the model adjusted for sociodemographic factors and lifestyle factors (model 3), the regression coefficients (95% confidence interval (CI)) were 0.00, 0.32 (−0.05–0.69), 0.44 (0.05–0.83), and 0.45 (0.02–0.87) across the quartiles of selenium intake. However, the association was attenuated and became marginally significant after further adjusting for BMI and hypertension (model 4). After the participants who only participated in one wave of the cognitive function test were excluded, high selenium intake was positively associated with global cognition score. Further adjusting for self-reported diabetes or stroke did not change the above association.

### 3.2. Association between Selenium Intake and Self-Reported Memory

Selenium intake was inversely associated with self-reported poor memory in a dose–response manner (Table 3). In the fully adjusted model, the odds ratios (ORs, 95% CI) across the quartiles of selenium intake were 1.00, 0.80 (0.67–0.96), 0.75 (0.61–0.91), and 0.68 (0.54–0.84), for self-reported poor memory and 1.00, 0.85 (0.73–1.01), 0.71 (0.60–0.85), and 0.65 (0.54–0.78), for self-reported memory decline.

### 3.3. Weight Status Modifies the Association between Selenium Intake and Global Cognitive Function

**Figure 2:**
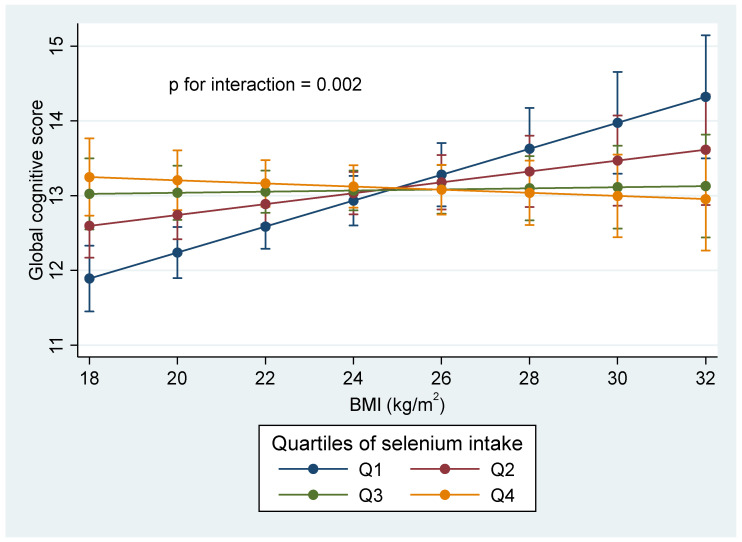
Interaction between selenium intake and BMI in relation to cognitive function score. Values are means (95% CI) derived by using the margins command in Stata after running a mixed linear model adjusted for age, gender, intake of energy and fat, education, urbanicity, smoking, alcohol drinking, physical activity, and fruit and vegetable intake. *p* for interaction between selenium intake and BMI was 0.002. An ordinal value (1, 2, 3, 4) was assigned to reflect the quartiles of selenium intake level. Q, quartile.

There was a significant interaction (*p* = 0.002) between selenium intake and BMI on the global cognitive function score (Figure 2). A strong positive association between selenium intake and good cognitive function was found among participants with low BMI. By contrast, there was an inverse association between selenium intake and cognitive function among participants with high BMI. In the subgroup analyses, a significant inverse association between selenium intake and a global cognition score < 7 was found only among participants with normal BMI (*p* for interaction = 0.012).

### 3.4. Subgroup Analyses

Selenium intake had no interactions with income, hypertension, gender, and urbanization in relation to the association with a global cognition score < 7. However, a regional difference in the association was observed. The association was only observed in the participants from the southern region but not those from the northern region (*p* for interaction = 0.005) (Table 4). 

The selenium-intake-to-iron-intake ratio was inversely associated with a global cognition score < 7. In the fully adjusted model, the ORs (95% CI) across the quartiles of the selenium-intake-to-iron-intake ratio for a global cognition score < 7 were 1.00, 0.88 (0.74–1.06), 0.62 (0.51–0.77), and 0.60 (0.47–0.77, *p* < 0.001). However, the association between selenium intake and cognitive function was independent of iron intake (data not shown).

## 4. Discussion

This study investigated the association of selenium intake with cognitive function in a large cohort of Chinese adults aged 55 years and above. Those with high selenium intake were associated with a high global cognition score and less likely to report poor memory and memory decline. Selenium intake had remarkable interactions with BMI and region. The positive association between selenium intake and cognition was only observed among those with a normal BMI and those who lived in the southern region.

Studies on the association between selenium status and cognitive function are inconsistent. In adults, selenium levels decrease with age and may contribute to age-related decline in cognitive function [29]. Using the National Health and Nutrition Examination Survey database, which focuses on adult participants aged 60 years or older, Li et al. found that high selenium intake is inversely associated with the prevalence of low cognitive performance [30]. A cohort study of 1666 French adults aged 60–70 years old indicated that those with low blood selenium levels have an elevated risk of cognitive decline [31]. A cross-sectional survey of 2000 Chinese rural residents aged 65 years or older in two provinces in China showed that lower nail selenium levels are substantially associated with lower cognitive scores [32]. A study conducted in old Sicilian individuals aged 60 years and above demonstrated that patients with AD have reduced blood selenium concentrations [33]. However, some studies found that plasma selenium is not associated with cognitive performance [34] and observed no changes in total selenium concentration in the serum or cerebrospinal fluid of participants with AD compared with patients with mild cognitive impairment and healthy controls [35]. Certain selenium species were even deemed risk factors for neurodegeneration in several observational studies. Vinceti et al. reported that higher levels of selenoprotein P in the cerebrospinal fluid are linked to a lower risk of sporadic amyotrophic lateral sclerosis (ALS), whereas elevated selenite in the cerebrospinal fluid is linked to a higher risk of neurodegeneration [36]. Recently, Vinceti et al. also observed an increased risk of developing AD later in life among patients with mild cognitive impairment who have increased concentration of the potentially toxic inorganic selenium species, selenate or Se(VI), in the cerebrospinal fluid [37].

The mechanisms linking selenium intake and cognitive function are yet to be fully elucidated. Oxidative stress is thought to be a major cause of cognitive impairment involved in the development of multiple neurodegenerative diseases [38]. Selenium is an important antioxidant; therefore, it may be especially helpful for combating oxidative stress. Free radicals are related with problems in mitochondrial function, neuroinflammation, synaptic transmission, and axonal transport; a shortage in this mineral may increase oxidative stress, which can lead to neuronal death [39,40]. The brain’s principal selenoproteins are selenoprotein P and glutathione peroxidase. Selenoprotein P is the major selenium transporter. This protein was found in the characteristic senile plaques and neurofibrillary tangles in the brains of patients with AD, which implies that it plays a key function in oxidative damage protection [41]. Glutathione peroxidase enzymes are expressed by neurons and glial cells [42]. They have a crucial antioxidant role; they accelerate the elimination of hydrogen peroxides, organic hydroperoxides, and lipid peroxides and protect cells from oxidative damage by lowering glutathione [43]. In summary, a selenium deficiency reduces the protective effects of antioxidant enzymes in the brain and causes oxidative stress, which can lead to brain tissue damage and cognitive decline.

A positive association between the ratio of selenium intake to iron intake and cognitive function means that higher selenium intake and lower iron intake lead to better cognitive function. Previous studies in China reported the negative effects of excessive iron intake on cognitive function in the elderly [44,45]. Furthermore, some population studies found that iron intake is positively associated with disorders that cause cognitive deterioration, such as PD [46,47]. Several human laboratory reviews also indicated a consistent correlation between iron deposition and cognitive impairment in humans with the usage of imaging techniques and found that iron chelation could be used as a potential therapy for memory deficits [48,49,50]. As a redox-active ion, iron impairs neurocognitive function by causing oxidative stress, damaging the mitochondria, and resulting in neurons’ energy production being hindered. Oxidative stress may also induce apoptosis, which contributes to neuronal loss and finally leads to cognitive decline [48].

In our study, the positive association between selenium intake and a low cognitive function score was only observed in the participants from the southern region, but not in those from the northern region. This finding could be attributed to the higher prevalence of dementia in North China compared with that in South China. The prevalence of dementia is 5.5% in North China and 4.8% in South China according to a meta-analysis published in 2018 [51]. The northern region may have more important factors influencing the prevalence of dementia other than selenium, such as obesity and smoking [2]. Additionally, the northern and southern regions of China have different dietary habits. For example, rice is the staple food in the south, whereas selenium-rich wheat is the staple food in the north, which means that the people from the north have a higher selenium intake than those from the south [52]. Some studies found that the dietary selenium intake of over 55 μg/day cannot improve selenoprotein synthesis or activity in selenium-deficient patients [53,54]. Therefore, no association in the north may also be due to excessive selenium intake in some participants from the north. However, we have some conflicting results here. For self-reported poor memory/memory decline, the inverse association with selenium intake was significant in the north (data not shown). Further studies are needed to elucidate the mechanisms of the regional difference in the association between selenium intake and cognition. 

The interaction between BMI and selenium intake with regard to cognitive function is interesting. The positive correlation between the cognitive function score and selenium intake diminishes as BMI increases. The relationship even becomes negative in obese participants. A study by Benjamin et al. indicated that cognitive decline slows down among patients with amnestic mild cognitive impairment (aMCI) and a higher BMI [55]. Another study found high BMI is associated with higher baseline impairment in aMCI and AD and slower clinical progression in aMCI [56]. Additionally, according to a cohort study of nearly 2 million people tracked retrospectively over 20 years, those who are underweight had a significantly increased risk of dementia [57]. These pieces of evidence imply that obesity may be an independent protective factor for cognitive impairment. The mechanisms of the interaction need to be further explored.

Several strengths in our study should be highlighted. First of all, this is a longitudinal study with multiple waves of dietary assessments based on three-day meal recalls and household food inventory records which provide a robust estimate of long-term selenium intake. Second, the large sample from different provinces allowed our findings to be generalized in a broader population. Third, we were able to control for a variety of potential confounders. Lastly, analyzing the link between high selenium intake and cognitive function was possible because of the large variation in selenium intake.

This study also has limitations. First, using a 3-day dietary intake survey to quantify long-term dietary selenium consumption may be insufficient. Second, respondents may have recall bias for the information collected on self-reported poor memory, memory decline, and other self-reported variables. Third, although we adjusted various sociodemographic and lifestyle factors in the multivariate analysis such as income, BMI, and diet, other residual confounding factors may also be present. Last, no data on selenium biomarkers (e.g., hair and serum selenium) were available.

## 5. Conclusions

In summary, high selenium intake was associated with a better cognitive function and lower risk of cognition decline in Chinese individuals aged 55 years and above regardless of gender, lifestyle, and sociodemographic factors. There was a significant interaction between selenium intake and iron intake, showing improved cognitive function in those with higher selenium intake and low iron intake. We also found selenium intake had remarkable interactions with BMI and region. Further studies with repeated measures of selenium intake and serum selenium in different populations are needed to confirm our finding.

## Figures and Tables

**Table 1:** Sample characteristics of Chinese adults aged ≥55 years old attending the first cognitive function test by quartiles of cumulative selenium intake (*n* = 4661).

	Q1	Q2	Q3	Q4	*p*-Value
	*n* = 1189	*n* = 1095	*n* = 1145	*n* = 1232	
Age (years)	65.9 (8.6)	63.7 (8.0)	62.1 (7.0)	62.0 (6.7)	<0.001
Sex					<0.001
Men	424 (35.7%)	456 (41.6%)	594 (51.9%)	763 (61.9%)	
Women	765 (64.3%)	639 (58.4%)	551 (48.1%)	469 (38.1%)	
Income					<0.001
Low	509 (43.3%)	355 (32.7%)	323 (28.3%)	276 (22.8%)	
Medium	362 (30.8%)	363 (33.4%)	391 (34.3%)	280 (23.2%)	
High	304 (25.9%)	369 (33.9%)	427 (37.4%)	652 (54.0%)	
Education					<0.001
Low	870 (85.9%)	811 (80.9%)	722 (67.9%)	645 (57.1%)	
Medium	80 (7.9%)	112 (11.2%)	206 (19.4%)	225 (19.9%)	
High	63 (6.2%)	79 (7.9%)	135 (12.7%)	260 (23.0%)	
Urbanization					<0.001
Low	425 (35.7%)	313 (28.6%)	264 (23.1%)	181 (14.7%)	
Medium	355 (29.9%)	326 (29.8%)	320 (27.9%)	297 (24.1%)	
High	409 (34.4%)	456 (41.6%)	561 (49.0%)	754 (61.2%)	
Region					<0.001
North	430 (39.9%)	367 (36.4%)	461 (45.6%)	658 (59.3%)	
South	647 (60.1%)	640 (63.6%)	551 (54.4%)	451 (40.7%)	
Smoking					<0.001
Non-smoker	845 (71.5%)	788 (72.2%)	749 (65.5%)	750 (60.9%)	
Ex-smoker	45 (3.8%)	38 (3.5%)	38 (3.3%)	49 (4.0%)	
Current smoker	292 (24.7%)	266 (24.4%)	357 (31.2%)	433 (35.1%)	
Survey year					<0.001
1997	611 (51.4%)	527 (48.1%)	486 (42.4%)	428 (34.7%)	
2000	189 (15.9%)	183 (16.7%)	180 (15.7%)	245 (19.9%)	
2004	271 (22.8%)	239 (21.8%)	282 (24.6%)	314 (25.5%)	
2006	118 (9.9%)	146 (13.3%)	197 (17.2%)	245 (19.9%)	
Alcohol drinking	272 (23.4%)	300 (27.8%)	388 (34.7%)	472 (38.8%)	<0.001
Physical activity (MET)	84.8 (100.4)	96.3 (105.5)	92.3 (101.2)	78.9 (89.4)	<0.001
BMI (kg/m^2^)	22.2 (3.7)	22.7 (3.6)	23.3 (3.5)	23.9 (3.4)	<0.001
BMI ≥24 (kg/m^2^)	293 (27.4%)	339 (33.0%)	426 (39.4%)	543 (47.2%)	<0.001
Energy intake (kcal/day)	1771.0 (520.8)	2025.6 (549.9)	2169.3 (587.5)	2393.8 (667.0)	<0.001
Fat intake (g/day)	51.2 (28.5)	62.5 (35.7)	69.0 (35.2)	83.2 (38.9)	<0.001
Protein intake (g/day)	47.5 (14.8)	59.2 (16.1)	66.7 (18.7)	80.2 (26.3)	<0.001
Carbohydrate intake (g/day)	277.2 (93.0)	301.6 (98.2)	313.3 (107.6)	321.4 (118.0)	<0.001
Iron intake (g/day)	15.5 (7.0)	19.0 (8.0)	20.9 (9.5)	25.1 (17.0)	<0.001
Intake of fruit (g/day)	13.1 (52.2)	18.3 (83.8)	21.6 (70.4)	38.9 (100.9)	<0.001
Intake of fresh vegetables (g/day)	249.2 (179.9)	270.3 (162.4)	281.3 (186.7)	298.2 (173.4)	<0.001
Intake of meat (g/day)	37.9 (49.5)	65.0 (67.2)	81.2 (77.6)	111.1 (104.5)	<0.001
Most recent selenium intake (mg/day)	21.6 (8.1)	31.8 (9.4)	39.6 (12.6)	62.5 (62.4)	<0.001
Cumulative selenium intake (mg/day)	22.3 (4.9)	32.5 (2.2)	40.3 (2.6)	60.7 (22.9)	<0.001
Hypertension	406 (37.0%)	332 (31.7%)	389 (35.6%)	439 (37.4%)	0.023
Diabetes	31 (2.7%)	29 (2.7%)	32 (2.9%)	57 (4.7%)	0.011
Stroke	28 (2.4%)	20 (0.9%)	19 (1.7%)	33 (2.7%)	0.29
Poor memory	350 (29.7%)	223 (20.7%)	210 (18.4%)	181 (14.8%)	<0.001
Memory decline	574 (49.3%)	446 (42.4%)	397 (35.7%)	362 (30.2%)	<0.001
Global cognitive function score <7	320 (26.9%)	215 (19.6%)	152 (13.3%)	150 (12.2%)	<0.001

Data are presented as mean (SD) for continuous measures, and *n* (%) for categorical measures.

**Table 2:** Association between quartiles of selenium intake and cognitive function among Chinese adults aged 55 years and above attending the China Health and Nutrition Survey (*n* = 4852).

	Quartiles of Selenium Intake							*p*-Value
	Q1	Q2		Q3		Q4		
Global cognitive function								
Model 1	0.00	0.80	(0.45–1.15)	1.35	(0.98–1.73)	1.80	(1.41–2.20)	0.000
Model 2	0.00	0.32	(−0.06–0.69)	0.44	(0.05–0.84)	0.46	(0.03–0.88)	0.037
Model 3	0.00	0.32	(−0.05–0.69)	0.44	(0.05–0.83)	0.45	(0.02–0.87)	0.044
Model 4	0.00	0.28	(−0.10–0.67)	0.37	(−0.03–0.78)	0.42	(−0.02–0.85)	0.075
Model 5	0.00	0.27	(−0.14–0.68)	0.29	(−0.15–0.72)	0.48	(0.01–0.95)	0.074
Model 6	0.00	0.29	(−0.12–0.70)	0.26	(−0.18–0.70)	0.50	(0.02–0.97)	0.071

Values are regression coefficients (95% CI) from mixed-effect linear regression. Model 1 adjusted for age, gender, and energy intake. Model 2 further adjusted for intake of fat, smoking, alcohol drinking, income (low, medium, and high), urbanicity (low, medium, and high), education (low, medium, and high), and physical activity level (continuous). Model 3 further adjusted for fruit and vegetable intake (continuous). Model 4 further adjusted for BMI and hypertension. Model 5 further excluded those who only participated in one wave of the cognitive function tests. Model 6 further adjusted for self-reported diabetes and stroke. All the adjusted variables are treated as time-varying covariates (except gender).

**Table 3:** Odds ratios (95% CI) for self-reported poor memory and self-reported memory decline by quartiles of selenium intake among Chinese adults aged ≥55 years in the China Health and Nutrition Survey (*n* = 4852).

	Quartiles of Selenium Intake							
	Q1	Q2		Q3		Q4		*p* for Trend
Self-reported poor memory								
Model 1	1.00	0.73	(0.63–0.85)	0.65	(0.55–0.76)	0.53	(0.45–0.62)	<0.001
Model 2	1.00	0.80	(0.68–0.94)	0.75	(0.63–0.90)	0.68	(0.56–0.83)	<0.001
Model 3	1.00	0.80	(0.68–0.94)	0.75	(0.63–0.89)	0.67	(0.55–0.81)	<0.001
Model 4	1.00	0.80	(0.68–0.95)	0.78	(0.65–0.94)	0.67	(0.55–0.83)	0.001
Model 5	1.00	0.81	(0.68–0.98)	0.76	(0.62–0.92)	0.68	(0.55–0.85)	0.001
Model 6	1.00	0.80	(0.67–0.96)	0.75	(0.61–0.91)	0.68	(0.54–0.84)	0.001
Self-reported memory decline								
Model 1	1.00	0.80	(0.67–0.96)	0.75	(0.61–0.91)	0.68	(0.54–0.84)	<0.001
Model 2	1.00	0.82	(0.72–0.93)	0.65	(0.57–0.74)	0.51	(0.45–0.59)	<0.001
Model 3	1.00	0.88	(0.76–1.02)	0.74	(0.64–0.87)	0.66	(0.56–0.78)	<0.001
Model 4	1.00	0.88	(0.76–1.02)	0.74	(0.64–0.87)	0.66	(0.56–0.78)	<0.001
Model 5	1.00	0.88	(0.75–1.02)	0.74	(0.63–0.87)	0.65	(0.54–0.77)	<0.001
Model 6	1.00	0.85	(0.73–1.01)	0.71	(0.60–0.85)	0.65	(0.54–0.78)	<0.001

Model 1 adjusted for age, gender, and energy intake. Model 2 further adjusted for intake of fat, smoking, alcohol drinking, income, urbanicity, education, and physical activity. Model 3 further adjusted for overall dietary patterns. Model 4 further adjusted for BMI and hypertension. Model 5 further excluded those who only participated in one wave of the cognitive function tests. Model 6 further adjusted for self-reported diabetes and stroke. All the adjusted variables are treated as time-varying covariates (except gender).

**Table 4:** ORs (95% CIs) for global cognitive scores < 7 across quartiles of selenium intake among Chinese adults aged ≥55 y by sample characteristics: China Health and Nutrition Survey (*n* = 4852).

	Quartiles of Selenium Intake								
	Q1	Q2		Q3		Q4		*p* Trend	*p* for Interaction
All	1.00	0.76	(0.63–0.92)	0.70	(0.57–0.87)	0.69	(0.54–0.87)	0.001	
Income									0.469
Low	1.00	0.66	(0.50–0.89)	0.64	(0.46–0.90)	0.48	(0.32–0.71)	0.000	
Medium	1.00	0.87	(0.63–1.20)	0.69	(0.49–0.99)	0.77	(0.51–1.17)	0.087	
High	1.00	0.85	(0.56–1.30)	0.82	(0.52–1.29)	0.96	(0.61–1.52)	0.930	
Overweight									0.012
No	1.00	0.69	(0.55–0.86)	0.60	(0.47–0.78)	0.52	(0.39–0.71)	0.000	
Yes	1.00	1.03	(0.72–1.47)	1.02	(0.71–1.46)	1.16	(0.79–1.71)	0.481	
Hypertension									0.680
No	1.00	0.80	(0.64–1.00)	0.67	(0.52–0.87)	0.70	(0.52–0.93)	0.003	
Yes	1.00	0.68	(0.49–0.94)	0.76	(0.54–1.07)	0.65	(0.44–0.96)	0.049	
sex									0.513
Men	1.00	0.90	(0.63–1.28)	0.69	(0.48–1.00)	0.67	(0.45–0.98)	0.019	
Women	1.00	0.70	(0.56–0.88)	0.71	(0.54–0.92)	0.72	(0.53–0.98)	0.016	
Urbanization									0.439
Low	1.00	0.66	(0.47–0.93)	0.66	(0.44–0.99)	0.62	(0.38–1.03)	0.022	
Medium	1.00	0.91	(0.64–1.29)	0.93	(0.63–1.38)	0.65	(0.41–1.02)	0.108	
High	1.00	0.75	(0.55–1.02)	0.60	(0.43–0.85)	0.74	(0.52–1.06)	0.064	
Region									0.005
North	1.00	0.90	(0.61–1.33)	0.79	(0.53–1.17)	1.26	(0.84–1.90)	0.385	
South	1.00	0.70	(0.55–0.89)	0.66	(0.50–0.88)	0.50	(0.35–0.72)	0.000	

Values are odds ratios (95% CI) from mixed-effect logistic regression. Mixed-effect logistic modes adjusted for age, gender, intake of energy and fat, smoking, alcohol drinking, income, urbanicity, education, physical activity, intake of fruits and vegetables, BMI, and hypertension. Stratification variables were not adjusted in the corresponding models. Income was categorized into low, medium, and high on the basis of tertiles of year-specific income.

## Data Availability

The datasets generated during and analyzed during the current study are available in the CHNS repository, https://www.cpc.unc.edu/projects/china accessed on 21 January 2022.
